# Decade‐Long Prodrome on Neuroimaging: Unique Insight into Probable Corticobasal Degeneration

**DOI:** 10.1002/mds.30245

**Published:** 2025-06-03

**Authors:** Xin You Tai, Pieter M. Pretorius, George K. Tofaris

**Affiliations:** ^1^ Nuffield Department of Clinical Neurosciences University of Oxford Oxford UK; ^2^ Department of Neurology John Radcliffe Hospital, Oxford University Hospitals NHS Foundation Trust Oxford UK; ^3^ Department of Neuroradiology John Radcliffe Hospital, Oxford University Hospitals NHS Foundation Trust Oxford UK

A 54‐year‐old man with a shaky hand was referred to Neurology clinic for possible Parkinson's disease. He had a prior history of nonfunctioning pituitary macroadenoma resected 13 years ago under surveillance with annual magnetic resonance imaging (MRI) scans and subsequent resection of residual tumor 2 years ago. No radiotherapy was administered due to the patient's choice. He described a 2‐year progressive jerky right hand tremor, which interfered with motor tasks such as typing and using chopsticks. His mobility was otherwise intact, being an avid cyclist with no falls reported. Work colleagues had noticed some word finding difficulties ascribed to anxiety. On further questioning, a history of behavioral changes emerged, leading to financial exploitation by a woman he met online and consequent marital difficulties. There was no topographical amnesia or hallucinations and no typical nonmotor symptoms of α‐synucleinopathy such as hyposmia, probable rapid eye movement (REM) sleep behavior disorder, or dysautonomia.

On examination, there were myoclonic jerks in the right hand at rest and dystonic posturing of the fingers (Video S1). With eyes closed, his right side demonstrated aimless movements suggestive of posterior‐variant alien limb phenomena. Reflexes were brisk on the right, including a pectoral jerk and a positive Hoffman's sign. There were sensory inattention, astereognosis, and apraxia to meaningless gestures. He scored 19/30 on the Montreal Cognitive Assessment (MoCA), with constructional apraxia, reduced fluency, and poor delayed recall.

Our diagnosis was corticobasal syndrome (CBS) that fulfilled the criteria for probable corticobasal degeneration (CBD), reflecting an underlying 4‐repeat tauopathy. Cerebrospinal fluid (CSF) analysis revealed a low Aβ42/40 ratio (0.044; <0.065 abnormal) but with normal tau levels (384 pg/mL; normal 146–595). Blood pTau‐217 levels were comparable to a cohort of frontotemporal dementia patients and healthy controls.^1^ Whole‐genome sequencing did not identify a genetic etiology.

MRI of the brain showed moderate global brain atrophy, with markedly asymmetrical atrophy of the superior parietal lobules and posterior frontal lobes with the left hemisphere worse affected (Fig. 1A, B). A similar distribution of hypometabolism was observed on positron emission tomography (Fig. 1C). We reviewed more than a decade of annual MRI brain imaging obtained due to surveillance of his pituitary tumor. This revealed progressive cerebral atrophy apparent up to 12 years before presenting to our clinic with disproportionate left parietal atrophy evident up to 7 years prior to presentation (5 years before symptom onset) (Fig. 1D). Hippocampal atrophy, which would suggest an (AD) Alzheimer's disease‐like underlying process,^1^ was not detected even on present‐day scans.

**FIG. 1:**
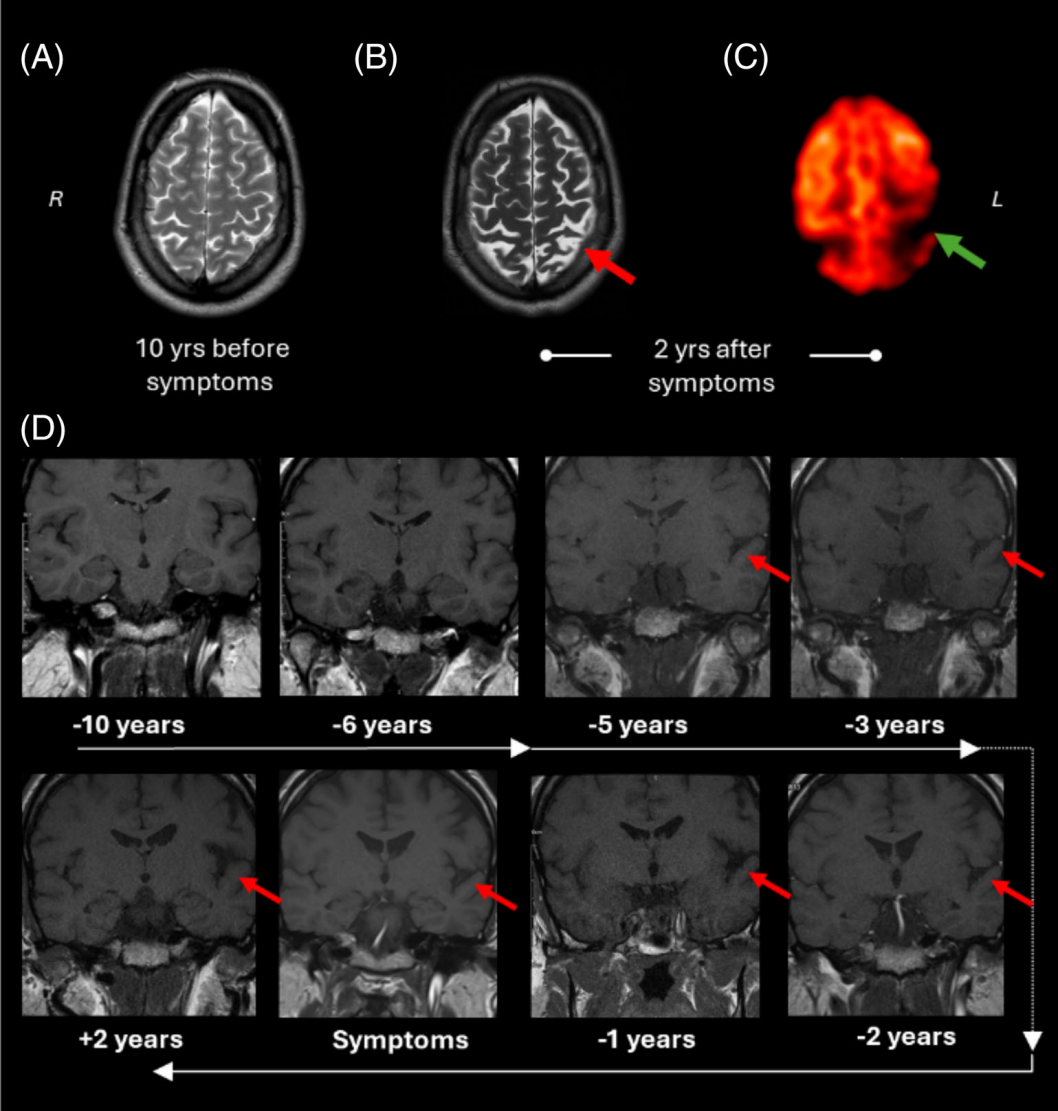
Brain MRI, axial T_2_‐weighted images, and axial 18F‐FDG PET images of the patient. (**A**) Mild generalized atrophy with a slight parietal predominance 10 years prior to symptom onset. (**B**) At the time of presentation to clinic, 2 years after symptom onset, there is significant atrophy with disproportionate, asymmetrical (left > right) parietal atrophy (red arrows). (**C**) Asymmetric hypometabolism in left parietal region at the time of presentation (green arrow). (**D**) A decade of annual coronal T_1_‐weighted images acquired to follow up the pituitary tumor show mild generalized atrophy 10 years prior to symptom onset with progressive volume loss. Asymmetric atrophy becomes evident up to 5 years before symptoms manifesting as widening of the left sylvian fissure on these images. MRI, magnetic resonance imaging; PET, positron emission tomography. [Color figure can be viewed at wileyonlinelibrary.com]

## Discussion

CBD is a rare neurodegenerative condition with an unclear natural history prior to diagnosis and a mean disease duration of 6 years.^2^ Neuropathological studies identified potential preclinical volume loss in anterior frontal and basal ganglia structures and gliotic changes in postmortem asymptomatic cases.^3^, ^4^ Uniquely, our patient had annual brain imaging for over 12 years prior to presentation, with the first scan demonstrating significant brain atrophy and a stepwise and lateralizing subsequent deterioration. This is the longest documented prodrome for probable CBD based on neuroimaging and offers unique insight into the neurodegenerative process, which is not typically described for a 4‐repeat tauopathy.

## Author Roles

(1) Research Project: A. Conception, B. Organization, C. Execution; (2) Statistical Analysis: A. Design, B. Execution, C. Review and Critique; (3) Manuscript Preparation: A. Writing of the First Draft, B. Review and Critique.

X.Y.T.: 1A, 1B, 3A.

P.M.P.: 1B, 3B.

G.K.T.: 1A, 1B, 3A.

## Supporting information


**Video S1.** Video demonstrating the relevant clinical signs.

## Data Availability

The data that supports the findings of this study are available in the supplementary material of this article.
